# Comparative analysis of the bioaccumulation of bisphenol A in the blood serum and follicular fluid of women living in two areas with different environmental impacts

**DOI:** 10.3389/fendo.2024.1392550

**Published:** 2024-10-08

**Authors:** Salvatore Raimondo, Maria Luisa Chiusano, Mariacira Gentile, Tommaso Gentile, Felice Cuomo, Raffaella Gentile, Domenico Danza, Laura Siani, Claudia Crescenzo, Mariangela Palmieri, Stefania Iaccarino, Mirella Iaccarino, Adriana Fortunato, Francesca Liguori, Antonio Esposito, Clelia Zullo, Loredana Sosa, Laura Sosa, Ida Ferrara, Marina Piscopo, Tiziana Notari, Raffaele Lacatena, Alberto Gentile, Luigi Montano

**Affiliations:** ^1^ Network for Environmental and Reproductive Health (Eco-FoodFertility Project), “Gentile S.A.S.” Research Center, Gragnano, Italy; ^2^ Department of Agricultural Sciences, University of Naples Federico II, Naples, Italy; ^3^ Residential Program in laboratory Medicine, Department of Medicine and Surgery, University of Milan Bicocca, Milan, Italy; ^4^ Mediterraneo Medical Assisted Procreation (MAP), Salerno, Italy; ^5^ Clinica Hera-Medical Assisted Procreation (MAP), Giugliano in Campania, NA, Italy; ^6^ Centro Megaride Fertility, Napoli, Italy; ^7^ Centro Fecondazione Assistita “Villa Bianca”, Napoli, Italy; ^8^ Centro Medical Assisted Procreation (MAP), ASL Napoli 2 Nord, Napoli, Italy; ^9^ Centro Embryos, Battipaglia, SA, Italy; ^10^ Medical Center “Gunè”, Acerra, NA, Italy; ^11^ Department of Biology, University of Naples Federico II, Naples, Italy; ^12^ Andrology Unit, Check-Up PolyDiagnostics and Research Laboratory, Salerno, Italy; ^13^ Andrology Unit and Service of Lifestyle Medicine in UroAndrology, Local Health Authority (ASL) Salerno, Coordination Unit of the Network for Environmental and Reproductive Health (Eco-FoodFertility Project), “St. Francis of Assisi Hospital”, Salerno, Italy; ^14^ PhD Program in Evolutionary Biology and Ecology, University of Rome “Tor Vergata”, Rome, Italy

**Keywords:** BPA, airborne bisphenol, pollution, follicular fluid, land of fires, environmental health, ART

## Abstract

**Introduction:**

Bisphenol A (BPA) is a common contaminant widely used in many industrial sectors. Because of its wide use and dispersion, it can be accumulated in living human bodies through both oral assumption and nondietary routes. BPA exhibits hormone-like properties, falling under the class of endocrine disruptors; therefore, it can alter relevant physiological functions. In particular, in women, it can affect folliculogenesis and therefore reproduction, contributing not only to infertility, but also to endometriosis and premature puberty.

**Methods:**

We conducted a multicenter study on 91 women undergoing a first *in vitro* fertilization (IVF) treatment in the Campania region (Southern Italy). We investigated the presence and concentration of BPA in serum and follicular fluids to assess the effects of airborne BPA contamination. The analysis was conducted on 32 women living in a low environmental impact (LEI) area, from the Sele Valley River and Cilento region, and 59 women living in a high environmental impact (HEI) area, the so-called “Land of Fires”, a highly contaminated territory widely exposed to illegal waste practices.

**Results:**

A higher average BPA content in both blood serum and follicular fluid was revealed in the HEI group when compared with the LEI group. In addition, we revealed higher average BPA content in blood serum than in folliclular fluid in the HEI area, with opposite average content in the two fluids in the LEI zone. In addition, our results also showed a lack of correlation between BPA content in follicular and serum fluids both in the overall population and in the HEI and LEI groups, with peculiar trends in different subsets of women.

**Conclusion:**

From our results, we revealed a heterogeneity in the distribution of BPA content between serum and follicular fluid. Further studies are needed to unravel the bioaccumulation mechanisms of BPA in highly polluted and nonpolluted areas.

## Graphical Abstract

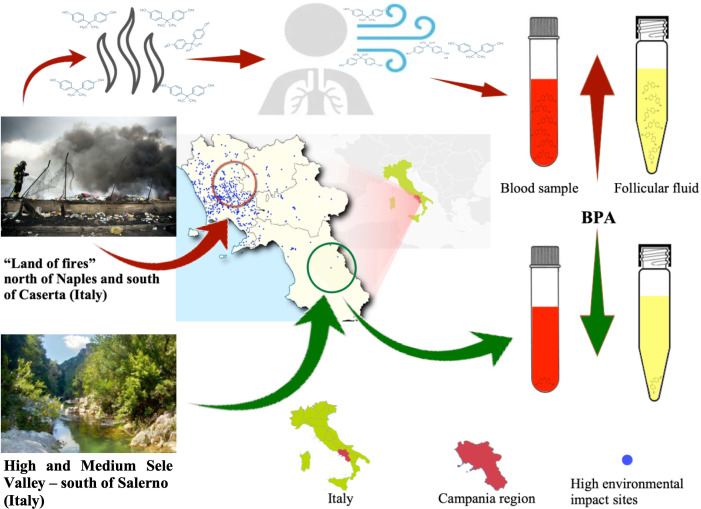

## Highlights

- Higher BPA levels are found in the blood and follicular fluid of women living in a polluted area
- Airborne bisphenol can strongly impact BPA accumulation in bodily fluids
- BPA bioaccumulation in blood serum and follicular fluid shows no correlation

## Introduction

Pollutants consist of various chemical substances and metabolites that may affect human health at different extents (1). Plastic derivatives in the environment are assumed to be increasing at an annual rate of 5% (2, 3). The development of recycling techniques for polymeric materials in recent years has not only contributed to an increase in their disposal, but also caused a considerable environmental impact involving the atmosphere (4, 5). Large quantities of plastics are disposed daily in landfills or burnt accidentally or intentionally, resulting in the release of volatile by-products into the atmosphere (2, 6, 7). Specifically, there is increasing attention on the levels of plastic derivatives in the air we breathe, probably due to the increasing knowledge on their possible impact on human health.

Bisphenol A (BPA), with a chemical formula of 4,4-dihydroxy-2,2-diphenylpropane, is a widely used substance, found as a raw material in the production of polycarbonate plastics and epoxy resins, key elements in varied industrial productions, such as food and beverage packaging, thermal paper (8), dentistry, and water pipes (9). Human beings can be exposed to BPA by food assumption and by non-dietary routes (10). Several authors speculate that oral exposure by food ingestion is the main source of BPA in humans in all age groups when not professionally exposed to this substance (11). The United States Environmental Protection Agency (EPA) has established a dose for humans (RfD) of 50 mg BPA/kg BW/day, based on toxicological studies (USEPA = United States Environmental Protection Agency, 2010), and the European Community has also adopted a similar metric. It is important to emphasize that the tolerable daily intake (TDI) usually considered assumes that oral intake is the main primary route of exposure. Dermal exposure and inhalation are not included, so the current TDI seems to be inadequate to suitably measure the real impact of BPA on human health. The environmental BPA pollution is therefore not adequately taken into consideration, although BPA concentrations in the environment have been extensively studied in aquatic and soil environments (12, 13), whereas studies on its presence in the air are still limited in number (14–16). Interestingly, recent studies highlight that BPA accumulated in the atmosphere can reach high concentration and can affect human health (17). Indeed, it is known that it is persistent in the environment due to continuous emissions (18) that allow its migration also in food (19), in the air (20, 21), in the skin (22), and in the blood (21). Measures of BPA bioaccumulation in the human body indicates that BPA is ubiquitous, and that it can be found in serum, amniotic fluid, urine, umbilical cord, blood, and placental tissues (23). Animal studies have shown that BPA exposure negatively affects the reproductive system (24). It is interesting to note that BPA was initially developed as a synthetic estrogen, but it was later proven to act as an endocrine disruptor. Indeed, it alters the endocrine system through binding to physiological receptors, such as estrogen receptors (ER) (ER1 and ER2), membrane-bound ERs, androgen receptors, peroxisome proliferator-activated receptor gamma, and thyroid hormone receptors (25). However, the genes activated by BPA differ from those triggered by estradiol (26).

Concerns about the effects of BPA exposure on reproductive health have led to numerous *in vitro*, animal, and human studies. BPA affects gametes by interfering with meiosis, folliculogenesis, and steroidogenesis. It also affects endometrial proliferation and endometrial receptivity (27, 28). The effects of BPA on human oocytes were also demonstrated (29). A significant increase in meiotic abnormalities due to casual exposure to BPA is the main indicator of adverse effects on oocyte development (30). Massive concentrations of BPA cause alterations in murine oocyte development, presumably because of oxidative stress, inducing increased meiotic arrest in germinal vesicles (GVs) or in metaphase I stages (31, 32).

Despite these lines of evidence, very little is known about the role of BPA as a reproductive toxicant in humans. One epidemiological study failed to prove an effect on human fertility (33); however, after controlled ovarian stimulation (COS), the level of urinary BPA appears to be inversely correlated with the number of oocytes retrieved. Indeed, as urinary BPA increases, the number of recovered oocytes decreases, on average by 12%, and the ovulatory peak of serum estradiol is reduced (34). In addition, urinary BPA appears inversely correlated with the number of mature oocytes after COS (35). Antral follicle count is a marker of ovarian reserve, a good predictor of ovarian response to COS, and it reduces significantly with increased urinary BPA (36). With regard to oocyte maturation (37), a negative dose–response correlation was observed between the rate of maturation of human oocytes *in vitro*, the percentage of bipolar spindles in the embryos, and the concentration of BPA added to the culture medium (38). Finally, a higher probability of embryo implantation failure was found in the presence of higher urinary BPA concentrations (39). However, it is not known whether these failures were of embryonic and/or endometrial origin.

Plasmatic components influence the production of follicular fluid (FF), which, in turn, is crucial for the development and vitality of oocytes (40, 41). Studies have shown that medical devices containing plastic can be a source of exposure to BPA in intensive care units (21, 42, 43). It is essential to assess chemical contaminants in assisted reproductive techniques to avoid exposure of gametes and embryos, e.g., BPA present in the needle tube for oocyte aspiration, in the FF collection tube, in Petri dishes for oocyte identification, and in the kit (syringe and catheter) for embryo or blastocyst transfer.

A study was conducted on 117 women with an average age of 34 years who underwent an assisted reproductive technology (ART) treatment. Blood serum showed increased levels of BPA correlating with an increased FF during oocyte pick-up (44). A study on 146 couples undergoing *in vitro* fertilization (IVF) found that BPA concentrations in various bodily fluids did not significantly impact reproductive parameters (45). In a cross-sectional study of 90 women undergoing IVF, higher BPA levels in FF were associated with more GV, while lower serum BPA concentrations were linked to more mature oocytes (29). A multicenter observational study involving 122 women undergoing PMA cycles found no association between BPA levels, oocyte quality, and pregnancy rate (46).

Despite the wide attention in assessing possible contaminations from BPA in blood serum and in FF in ART, only few human studies have focused on measuring BPA in fluids in association with different environmental conditions and pollution (44, 47–49).

Regardless of the large amount of effort exerted to improve environment quality, environmental pollution remains a significant problem and represents a relevant health risk on reproductive health. Heavy metals, polycyclic aromatic hydrocarbons (PAHs), polychlorinated biphenyls, dioxins, pesticides, and ultra-fine particles severely impair the whole human defense system mechanisms. For example, the correlation between heavy metals and oxidative DNA damage has been widely discussed (50). Indeed, some heavy metals can change the properties of the sperm nuclear basic proteins, which can change their canonical ratios and their protective role on DNA, being involved in oxidative DNA damage (51), as demonstrated in areas with a high environmental impact. It has also been shown that there is a lower seminal antioxidant activity in spermatozoa of residents in polluted areas (52). The phenomenon is so significant that reproductive system components have been considered important biosensors of environmental pollution (53). This is because, in areas with greater environmental impact, the altered environmental conditions, together with direct and indirect short- and long-term effects, and also due to other adverse events, such as viral infections, could cause a deterioration in sperm quality with important consequences on male fertility (54–56).

Environmental pollution also has a strong impact on the female reproductive domain. In this regard, it was recently shown that kallikrein bound to serine peptidase 3 could be an early biomarker of environmental exposure on the immune and reproductive systems of young women (57).

In this paper, we report the results of a specific research on women in the context of the EcoFoodFertility research project (www.ecofoodfertility.it). We evaluated BPA levels in blood and FF in a population of women, permanently living in two areas with different environmental impacts in the Campania Region (Southern Italy), who underwent an ART cycle for the first time. The two residential areas from which the recruited women were selected are called the “Land of Fires”, straddling the provinces of Naples and Caserta, an area territorially known for the illicit disposal and incineration of toxic and urban waste. These illegal practices have significant consequences on the health of the local population (58, 59), and the area is therefore classified, according to a mathematical model developed by a multidisciplinary team, as a high environmental impact (HEI) area (60). The second area is the Sele Valley, a low environmental impact (LEI) area located in the province of Salerno. More specifically, there are regions in the world where the practice of burning plastic waste is prevalent, such as certain areas in India where the highest levels of bisphenol in the air have been measured. A positive correlation between BPA and 1,3,5-triphenylbenzene, a tracer for plastic burning, has been identified in these regions. This correlation highlights that the open burning of plastic waste is a significant source of BPA emissions into the atmosphere (6, 7). Consequently, it is highly plausible that the widespread and illicit incineration of waste containing amounts of plastics, so pervasive in the “Land of Fires”, may represent a substantial source of human contamination via inhalation.

Our study reveals significant differences in the bioaccumulation of BPA in blood serum and FF, which is higher, on average, in women living in an HEI area than in those living in an LEI area. In addition, we revealed peculiar trends showing a heterogeneous level of BPA in different subsets of women and therefore the need for further studies to unravel the mechanisms of the bioaccumulation of BPA in the bodily fluids of women in highly polluted and nonpolluted areas.

## Materials and methods

### Cohort selection

The present work is part of the EcoFoodFertility research project (www.ecofoodfertility.it, approved by the Ethical Committee of the Local Health Authority Campania Sud-Salerno, Committee code n. 43 of 30 June 2015).

We selected 91 women with normal ovarian reserve, aged between 26 and 47 years [34 (mean) ± 4 (SD)] with a history of infertility ranging from 26 to 39 months, who underwent a cycle of ART treatment for the first time, to evaluate BPA levels in blood and FF.

The women were from two residential areas in the Campania Region (Southern Italy), where they permanently lived (at least 5 years): the “Land of Fires” straddling the provinces of Naples and Caserta, an area territorially known for the illicit disposal and incineration of toxic and urban waste, classified, according to a mathematical model developed by a multidisciplinary team, as an HEI area (60). The second area is the Sele Valley, an LEI area located in the province of Salerno.

The women were divided into two groups according to living area. The LEI group consisted of 32 women (**Supplementary Table 1**, numbered from 1 to 32) selected from the LEI, with normal ovarian reserve inferred by the anti-Müllerian (AMH=3 ± 2 ng/ml) (61–63), and the HEI group is composed of 59 women (**Supplementary Table 1**, numbered from 33 to 91) living in the HEI area, with normal ovarian reserve (AMH=2 ± 2 ng/mL).

### Metadata and variable description

Metadata concerning the participants were collected through a “clinical-anamnestic form” in which they reported about their health conditions, as well as use and/or abuse of alcohol, smoking, and, in some cases, previous pregnancies. Participants have no other severe chronic diseases beyond those, if any, associated with gynecological aspects. They have been living in their respective selected areas for at least 5 years; they are not professionally exposed to risk factors and have not taken contraceptive pills for at least 2 years. They deny having used drugs over the 12 months before serum collection and oocyte pick-up. Participants were asked about age at menarche and whether they had experienced previous spontaneous and/or voluntary abortions. All participants have a regular menstruation cycle; the cycle length varies between 28 and 30 days, regular for length, rhythm, and volume. Moreover, body mass index (BMI), waist–height ratio (WHR), and the Ferriman–Gallwey (FG) score for hirsutism were also determined. BMI classes were defined according to the World Health Organization (WHO) obesity levels.

Health conditions are described in terms of ovulation regularity, ovarian reserve (OR), possible endometriosis, and/or polycystic ovary syndrome (PCOS). FG score values range from 1 to 4 according to Ferriman and Gallwey (64, 65).

Smoking attitudes are indicated with “yes/no/p.h.” (previous history of smoking) values. No previous pregnancy (nulliparous) variables are indicated with “yes/no” values.

The fertilization rate (FR) indicates possible events of previous assisted reproductive technologies (e.g., IUI or IVF) or miscarriages (spontaneous or voluntary); classes were defined as follows: 0 for no previous events; 1 for one event of assisted fertilization or spontaneous miscarriage; 2 for at least two events of assisted fertilization or spontaneous miscarriage.

**Table 1** summarizes the categorical variables, their classes, and the population distributions. Numerical variables' summary statistics are indicated in **Table 2**.

**Table 1:** Metadata description, variable classes, and sample size (total and distribution per zone).

Variable	Class	Total	Size per zone	
			HEI	LEI
**Population**		91	59	32
**Age_Groups**	26 ≤ *x* ≤ 30	21	13	8
	31 ≤ *x* ≤ 34	41	25	16
	35 ≤ *x* ≤ 47	29	21	8
BMI_Groups*	19 ≤ *x* ≤ 24	44	32	12
	25 ≤ *x* ≤ 30	41	21	20
	31 ≤ *x* ≤ 38	6	6	0
**Health_Conditions**	Normovulatory	56	34	22
	norm_OR	6	2	4
	Oligomenorrhea	11	9	2
	poor_OR	2	2	0
	PCOS	11	8	3
	Endometriosis	5	4	1
FG_score***	1	47	20	27
	2	39	34	5
	3	5	5	0
**Smoker**	Yes	10	8	2
	No	65	40	25
	p.h.	16	11	5
**Alcohol**	Yes	24	16	8
	No	67	43	24
**Nulliparous**	Yes	80	54	26
	No	11	5	6
FR***	0	26	7	19
	1	44	32	12
	2	21	20	1

norm_OR, normal ovarian reserve.

poor_OR, reduced ovarian reserve.

p.h., previous history (of smoking).

PCOS, polycystic ovary syndrome.

Statistically significant differences by ChiSq tests in the population size between

zones per class in each variable are indicated (*p<0.05: ***p<0.001).

**Table 2:** Summary statistics in total and per zone.

Variable	Min	Total	Max	Min	HEI	Max	Min	LEI	Max
		Mean(SD)			Mean(SD)			Mean(SD)	
Seric BPA***	1.5	65(49)	182.4	3.1	96(27)	182.4	1.5	7(15)	86.1
Follicle BPA***	4.2	21(15)	81.5	4.2	25(16)	81.5	5.8	13(8)	35.6
FSH***	0.98	7(3)	18.3	2.6	8(3)	16.2	0.98	4(3)	18.3
E2***	10.2	54(30)	124.2	10.2	66(30)	124.2	12.5	33(17)	80.3
**AMH**	0.02	3(2)	12.4	0.02	2(2)	12.4	0.09	3(2)	8.49
WHR**	0.4	0.75(0.01)	1	0.4	0.77(0.13)	1	0.6	0.72(0.06)	0.8
**BMI**	20.7	25(3)	38	21	26(3)	38	20.7	25(2)	28.2
**Age**	26	34(4)	47	27	34(4)	45	26	33(5)	47

Statistically significant differences of means between HEI and LEI zones are indicated

as **p<0.01; ***p<0.001 (Wilcoxon test, two-tailed).

### Determination of hormones and BPA

To determine the concentrations of hFSH, hLH, and estradiol, an immunoassay in chemiluminescence with paramagnetic particles is used, using the Access 2 Immunoassay System by Beckman Coulter according to the manufacturer’s instructions. The values are expressed as follows: hLH and hFSH in mIU/mL, and estradiol in pg/mL.

For the quantitative measurement of circulating AMH, the ELFA (enzyme-linked fluorescent assay) technique is used, with an automatic VIDAS analyzer from BioMerieux-France, according to the manufacturer’s instructions. Values are expressed in ng/mL.

For the BPA assay, an enzyme-linked immunosorbent assay was used, and values are expressed in ng/mL (Booster Biological Technology, 3942 B Valley Ave, Pleasanton, CA 94566). For all samples, BD Vacutainer®-treated glass-reinforced USP Type III, non-siliconized, 5-mL tubes with a red cap, without additives and kept in a horizontal position, were used. The FF processed were bloodless with a negative albumin test.

### Statistical analysis

The statistical significance of differences in means defined in the two regions under investigation for any of the numerical variables was evaluated by a two-tailed Wilcoxon test, while a chi-square test of independence was used to determine whether categorical variables are likely to be related or not to living area (McHugh ML. 2013).

### Clustering

In order to discover groups of patients with a similar content of BPA in fluids, we performed a K-means clustering (K was set to 6, according to the corresponding figure of merit, not shown). Mean normalization and scaling were applied as a preprocessing step in order to account for variance inequalities per variable. Metadata description is schematized in the clustering per patient according to the classification of each variable.

Pearson correlation analyses were performed in order to assess the relationships between serum and follicular BPA contents and among all numerical physiological variables. This was done for the whole population (HEI+LEI) and stratified per area (HEI or LEI) and per cluster group.

## Results

### Cohort variables per area

**Table 1** shows the data organization for the sampled population with the total distribution per variable and per area (HEI or LEI), evaluating statistical significance of differences per area by a chi-square test. The sampled population results are not uniformly distributed for BMI, FG score, and FR (risk of fertility) variables. These differences may reflect a bias in the sampling or effects of the specific environmental context.

**Figure 1:**
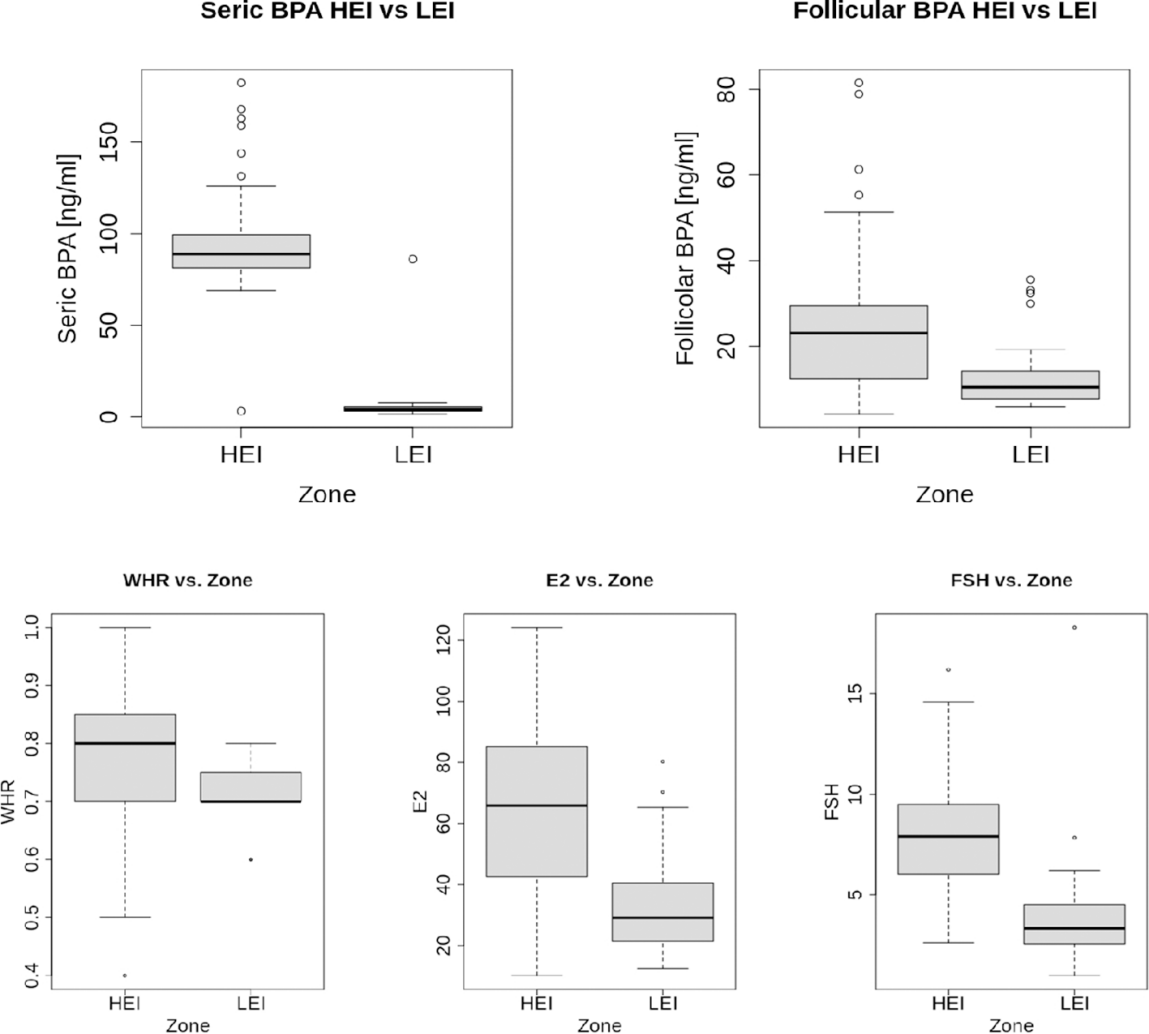
Boxplots of numerical variables that resulted with significant differences in means per zone (two-tailed Wilcoxon test shown in **Table 2**).

**Table 2** shows summary statistics of the numerical variables representing physiological parameters together with mean and standard deviation (SD) values for age and BMI in total and per zone. Statistically significant differences in means between the HEI and the LEI populations occur in BPA, in FSH, in E2 contents (*p*<0.001) and in WHR (*p*<0.01). All these variables show higher mean values in the HEI population than in the LEI. It is noteworthy that the serum BPA content shows striking differences in the two zones (**Figure 1**).

**Table 3** indicates summary statistics per serum and follicle BPA in total, per zone, and stratified per age and per BMI, since these two variables may be confounders in the assessment of the BPA content in the human body (**Supplementary Figure 1**). The stratification shows that age and BMI in the two populations do not affect the statistical significance of the difference in means of the BPA content in the two areas for both tissues. In addition, average BPA values also show opposite average trends in the two areas: the mean BPA content in blood serum is significantly higher than that in the FF of people from the HEI area, while, although with lower discrepancy, FF BPA is significantly higher than blood serum content (*p*<0.001 by a two-tailed Wilcoxon test) in people from the LEI area. Last, no significant correlation was found among numerical variables and BPA contents in each tissue (details not shown).

**Table 3:** Summary statistics of BPA in seric and follicular fluids stratified per age and BMI groups (total and per zone).

Variable	Class	Mean(SD)		
		Total	HEI	LEI
**Seric BPA**	26 ≤ age ≤ 30***	58(39)	85(8)	15(29)
	31 ≤ age ≤ 34***	62(50)	98(24)	4(24)
	35 ≤ age ≤ 47***	74(54)	101(37)	4.3(1.3)
**Follicle BPA**	26 ≤ age ≤ 30*	16(8)	19(7)	12.4(7.6)
	31 ≤ age ≤ 34**	19(13)	23(14)	12(14)
	35 ≤ age ≤ 47	27(20)	30(21)	17(11)
**Seric BPA**	19 ≤ BMI ≤ 24***	74(49)	101(27)	3.9(1.4)
	25 ≤ BMI ≤ 30***	50(48)	90(30)	8(18)
	31 ≤ BMI ≤ 38	94(20)	94(20)	/
**Follicular BPA**	19 ≤ BMI ≤ 24**	23(18)	26(20)	12(8)
	25 ≤ BMI ≤ 30***	19(11)	24(11)	14(8)
	31 ≤ BMI ≤ 38	20(10)	20(10)	/

Statistically significant differences by the Wilcoxon test (two-tailed) between HEI

and LEI zones are indicated (*p<0.05; **p<0.01; ***p<0.001)./ indicates no people from the specific zone.

### Clustering in terms of BPA content in the fluids

**Figure 2:**
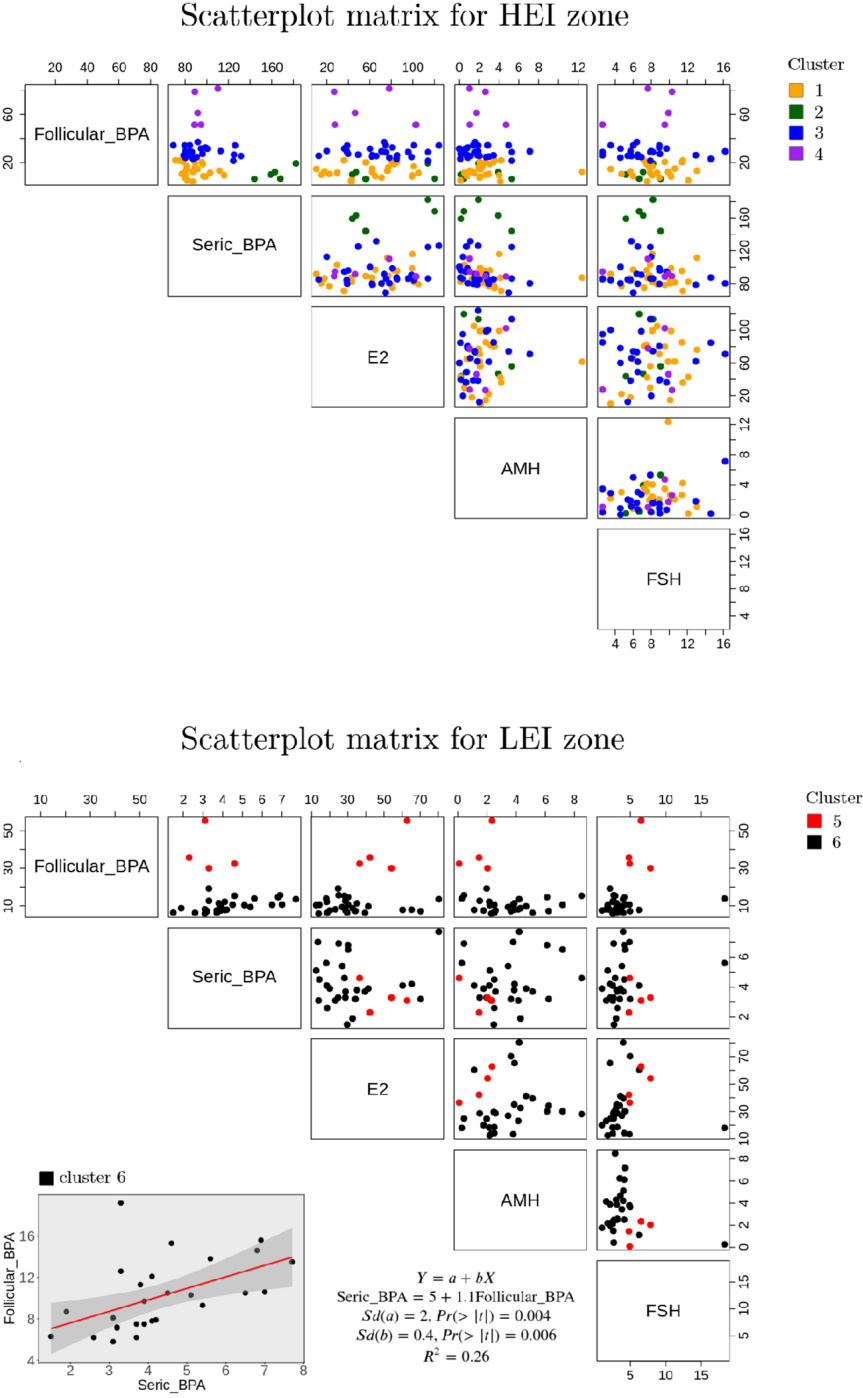
Scatterplot matrices of the principal parameters considered per cluster in the LEI and HEI areas.

**Figure 3:**
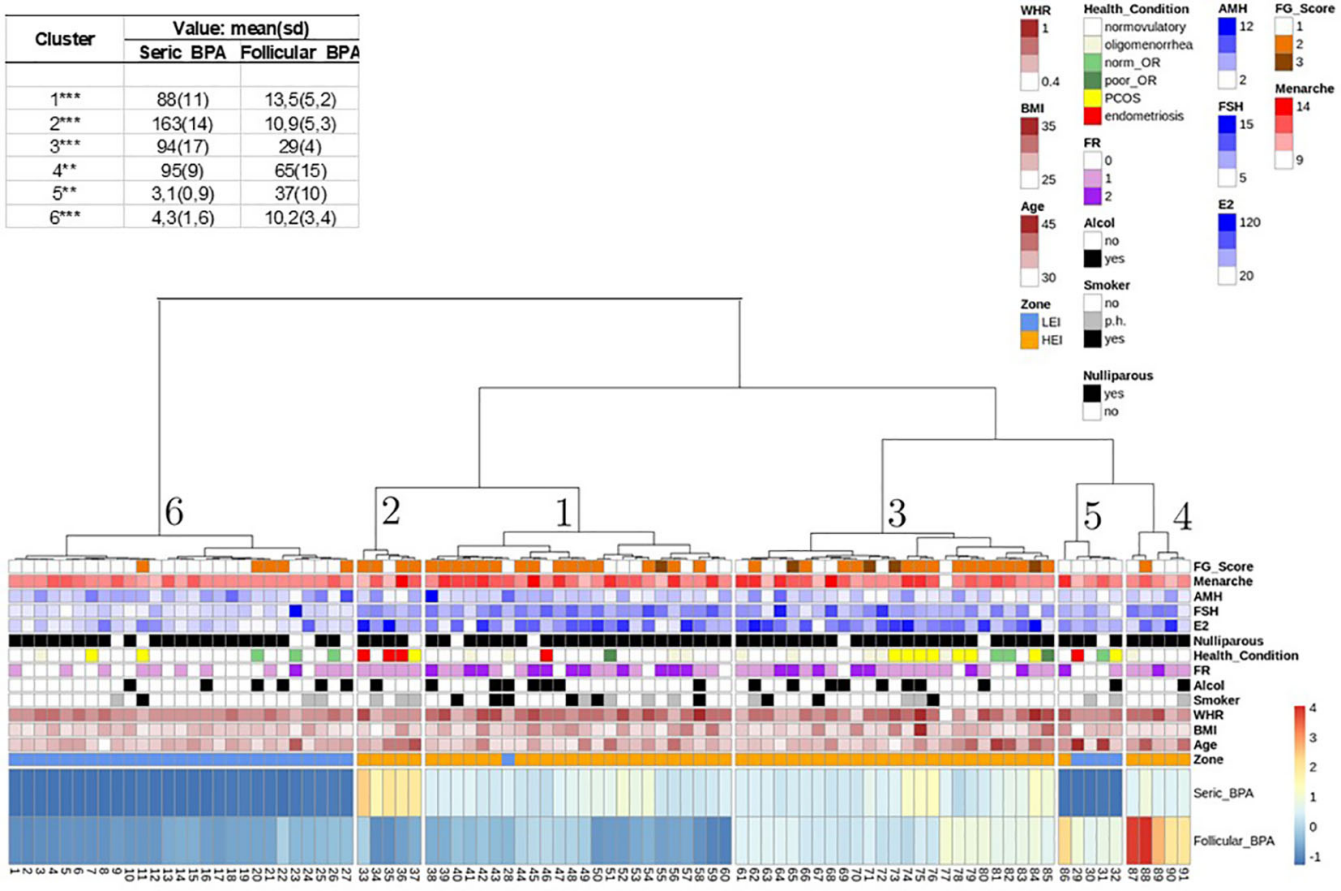
Hierarchical K-mean clustering on seric and follicular fluid BPA. All other variables and their range of variability are reported in the legend. Means and statistical significance between HEI and LEI zones are also indicated as *p<0.05:**p<0.01;***p<0.001 (two-tailed Wilcoxon test).

The unsupervised hierarchical clustering based on a K-means approach on PBA contents in blood serum and FF on the entire sample of 91 individuals is shown in **Figure 2** (list of individuals per area, BPA levels, and distribution per cluster are also reported in **Supplementary Table 1**). Statistics on the BPA content per cluster are also summarized in **Figure 3**.

Cluster 6 includes the majority of individuals from the LEI area (27 out of 32 women). Cluster 5 is also composed mainly of LEI individuals; specifically, four individuals are from the LEI area and one (individual number 86) is from the HEI area. Cluster 1 (24 individuals), cluster 2 (5 individuals), cluster 3 (25 individuals), and cluster 4 (5 individuals) are all from the HEI area with the only exception of individual number 28 in cluster 1, who is reported to be from the LEI area. It is evident that the BPA compositional features of the serum for individuals 28, classified as belonging to the LEI area, and 86, classified to be from the HEI area, are atypical in comparison with people from the same area, reflecting a higher similarity to people living in the other area, respectively. This explains why these two individuals fall under two clusters composed of people from the other area. This also confirms that blood serum BPA appears to be a major indicator of the living area with only two exceptions. Interestingly, in a second verification from the medical doctor, it was revealed that individual 28 was indeed living in the HEI zone.

Cluster 6 patients are those with the lowest BPA content in both follicular and serum fluids (as shown in **Figure 3**), when compared with the other clusters from the analysis. Moreover, on average, the follicle BPA content is significantly higher than the serum content, confirming the general average trend presented for people in the LEI area (**Table 2**). Cluster 5 includes only five individuals, four from the LEI area and 1 from the HEI area. This group is also characterized by a higher BPA follicle content than serum fluid content (as shown in **Figure 3**). However, it should be emphasized that the BPA content in the follicle in this cluster is three times higher than the average PBA content of the LEI population, indicating a peculiar PBA content in FF for this cluster of people. Interestingly, all members of this population suffered from allergies and were exposed to cortisone treatments before the pick-up (personal communication with a medical doctor).

Clusters 1, 2, 3, and 4, mainly representing people from the HEI area, show a higher BPA content in serum than in FF, with the highest value in cluster 2. Notably, this is the cluster with the lowest average BPA content in the follicle among people from the HEI area (**Figure 2**). Interestingly, four out of five individuals in cluster 2 show pathologies in the reproductive systems with endometriosis (one has polycystic ovary). cluster 4 has the highest BPA content in FF in the overall dataset, although no peculiar trends in BPA content are revealed in serum.

The variability of the relative BPA content in the two fluids in both HEI and LEI areas revealed by the cluster analysis further confirms the lack of correlation between BPA contents in blood serum and FF, as it was revealed by the higher BPA content in blood serum when compared to FF in the HEI area, and the lower BPA content in blood serum when compared to FF in the LEI area (**Table 3**), highlighting the peculiar bioaccumulation in the two populations as well as in the different clusters of individuals, although confirming the main average trends in the areas, i.e., a higher average BPA content in the HEI area in both blood serum and FF with respect to the LEI area (**Table 3**).

To further investigate this heterogeneity in relative content, we also evaluated if any significant linear correlation existed between blood serum and FF BPA, and among these variables and other numerical variables, in total, in the HEI and LEI populations, and in a larger cluster (1,3,6) (**Figure 2**). We did not detect any significant correlation among numerical variables showing significant differences in **Table 2** (results not shown). BPA content in the two fluids in each cluster showed a weak linear correlation only in cluster 6 (**Figure 2**).

## Discussion

Only few experimental studies have assessed the bioaccumulation of BPA in FF. Consequently, there is limited evidence regarding the potential direct effect of BPA on ovarian sexual steroid synthesis (38, 48, 66). *In vivo* and *in vitro* studies using murine models have reported conflicting associations between higher BPA exposures and reduced steroidogenic enzyme activities in the ovarian theca (23, 67) and in granulosa cells (58, 66, 68). This discrepancy may explain the negative impact on oocyte quality. While some studies have reported null or inconsistent results (69–71), more recent scientific evidence suggests that high levels of BPA in FF can negatively impact pregnancy rates (72, 73). However, the effective dose of unconjugated BPA needed to disrupt estrogen synthesis in human populations may not align with general levels of “baseline” exposure, mainly through dietary sources. The clinical impact of this disruption remains unclear. Nonetheless, susceptible subgroups or those with unusually high exposure to BPA, as seen in HEI areas, may be considered vulnerable populations (74).

The ovarian blood–follicle barrier (BFB) likely plays an important but still poorly understood role in this process. The BFB is crucial for preventing the entry of foreign or harmful substances (toxics substances, drugs, etc.) into the ovary. It regulates the composition of FF during follicle development and filters various components based on the needs of folliculogenesis (e.g., proteins, peptides, electrolytes, ions, and sugars). This barrier is nonselective for certain drugs and/or chemotherapy. One example is doxorubicin, an anticancer drug, which can penetrate the BFB and induce ovarian failure, leading to oocyte apoptosis (75, 76).

Our multicenter study aims to monitor BPA contamination in the serum and FF of women undergoing oocyte pick-up for IVF in two different geographical areas with a different environmental impact as part of the EcoFoodFertility project (www.ecofoodfertility.it). The occurrence of different environmental sources of BPA contamination (hazardous sites near living places such as deliberate burning of plastics, exposure or use of potentially toxic agents, consumption of local food, etc.) suggests that individuals permanently residing in HEI areas are at a higher risk of BPA exposure. Our results, comparing samples from women permanently living for at least 5 years in one of two areas, a very high polluted area of the Campania region (HEI), the so-called “Land of Fires”, due to toxic plastic waste and its illegal vandalistic combustion, and an LEI area, which includes the Sele Valley River and the Cilento regions in the province of Salerno, confirm this hypothesis, with high average differences in BPA concentrations in blood serum and FF in the HEI and LEI populations.

Patients in the HEI area show notably higher BPA levels in both tissues compared to those in the LEI area. The significant differences in serum concentrations between the two groups can be explained by also considering numerous studies in mouse and human models that hypothesize epithelial damage of membrane proteases or the targeted deletion of both structural and junctional barrier genes that alter permeability (77). BPA is acknowledged as a xenoestrogen and is responsible for the permeability of the hemato-alveolar and hemato-gastric membrane directly proportional to its concentration (78). Although some studies show that exogenous proteases alone can disrupt the ovarian BFB, this does not fully explain why it also occurs in a proportion of the population living in the same area (79). The variability in BPA concentrations in FF between and within the two groups of women therefore prompts further investigation.

The additional significant finding reported here is that patients in the HEI area have a considerably higher BPA content in FF compared to participants in the LEI group, but also that the LEI group has a higher BPA content in the follicle than in the serum. In general, there is a lack of correlation between BPA contents in serum and FF, that could be interpreted as a different permeability and/or impermeability of the ovarian hemato-follicular membrane as an effect of exposure not only to BPA but also to other harmful factors. Recent studies have shown that this alteration occurred in patients with PCOS (80). The presence of a weak correlation between BPA in blood serum and FF in the LEI population in cluster 6, grouping only people from the LEI area, does not appear to be a general rule in our sampling, highlighting that lower contamination levels follow the expected trends of bioaccumulation suggested in previous similar efforts that addressed the correlation between blood serum BPA levels and FF (29).

In addition, the presence of two clusters of people in the LEI zone reveals that alterations in FF BPA levels can be due to additional factors influencing or related to health conditions, such as the reported allergies (personal communication with doctors) for people in cluster 5. Indeed, it is known that allergies are associated with BPA exposure (81, 82) and that BPA bioaccumulation is associated with immune-related diseases (83, 84).

No relevant feature emerging from the associated metadata based on the information collected from the sampled populations in our dataset was useful to consistently explain the different clusters of people in the HEI area, with the only exception of cluster 2, where the highest BPA content in blood serum in the overall group of people is associated with four out of five individuals who manifest alterations of tissues from organs of the reproductive system because of endometriosis (three out of five individuals) and PCOS (one out of five individuals) as discussed in other studies (72, 85, 86). Nevertheless, PCOS-affected women in our sample, although more frequent in the HEI area, do not appear to be significantly over-represented in this area, neither are they associated with BPA content in the two fluids, in relation with other previous lines of evidence (87).

It is evident from our analysis that further sampling and deeper molecular analyses would be necessary to check on these aspects and confirm our evidence. Indeed, our results are clear in distinguishing BPA content in fluids from people living in the two different areas, but further investigations are needed to fully unravel the bioaccumulation mechanisms of BPA in blood serum and FF and to explain the BPA absorption and consequent health damage. Nevertheless, the results of this study indicate that the higher bioaccumulation in the HEI group of women appears to be associated withare different environmental conditions of the areas under investigation and with comparative studies already carried out within the EcoFoodFertility project on the male side. Indeed, it provides further evidence that pollution in this area is not just a reproductive risk (80, 88–93). Of course, molecular analyses will be needed to investigate the effects of BPA in more detail, just as we looked for the effects of heavy metals and VOCs in human sperm (94, 95).

## Conclusions

Our analyses revealed significant differences in BPA concentrations in the serum and FF of women undergoing an IVF program in geographical areas with different environmental impacts. The presence of various environmental sources of BPA pollution, particularly due to the widespread incineration of toxic and municipal waste containing a significant amount of plastic, would entail a higher risk of human contamination by high atmospheric levels of BPA and other contaminants for women residing in the HEI area. Considering the inhomogeneity of BPA concentrations in the FF between the two groups and in the same groups, and the lack of overall correlations between the different groups and subgroups, they deserve more in-depth investigations. Although many studies demonstrate that exogenous proteases (including BPA) are sufficient to deteriorate the epithelium of the BFB, this does not fully explain why this occurs at different extents in the population of the same area. The lack of correlation between BPA values between serum and FF should be interpreted as different permeability and/or impermeability of the ovarian blood–follicular membrane, which can be hypothesized to be associated with specific pathological conditions (PCOS and endometriosis). From the current study, the presence of BPA in the FF of infertile women undergoing IVF reveals a presumable negative impact on pregnancy rates as well.

Further biomonitoring studies with larger sample size and integration with additional biomarkers and metadata collections, possibly associated with more in-depth examination of the cellular and molecular assets in patients, are essential to understand the mechanisms that regulate the presence of BPA and/or its metabolites, and undertake territorial reclamation to protect public health.

## Data Availability

The original contributions presented in the study are included in the article/**Supplementary Material**. Further inquiries can be directed to the corresponding author.

## Supplementary material

Supplementary Figure 1 Boxplots of numerical variables that resulted with significant differences in means, stratified per Age and BMI per zone (two tailed Wilcoxon test shown in **Table 2**).
