# Supplementary figures and images for: Comparative analysis of the bioaccumulation of bisphenol A in the blood serum and follicular fluid of women living in two areas with different environmental impacts
Source: Front Endocrinol (Lausanne). 2024 Oct 8;15:1392550. doi: 10.3389/fendo.2024.1392550 (PMC11495266; doi:10.3389/fendo.2024.1392550)

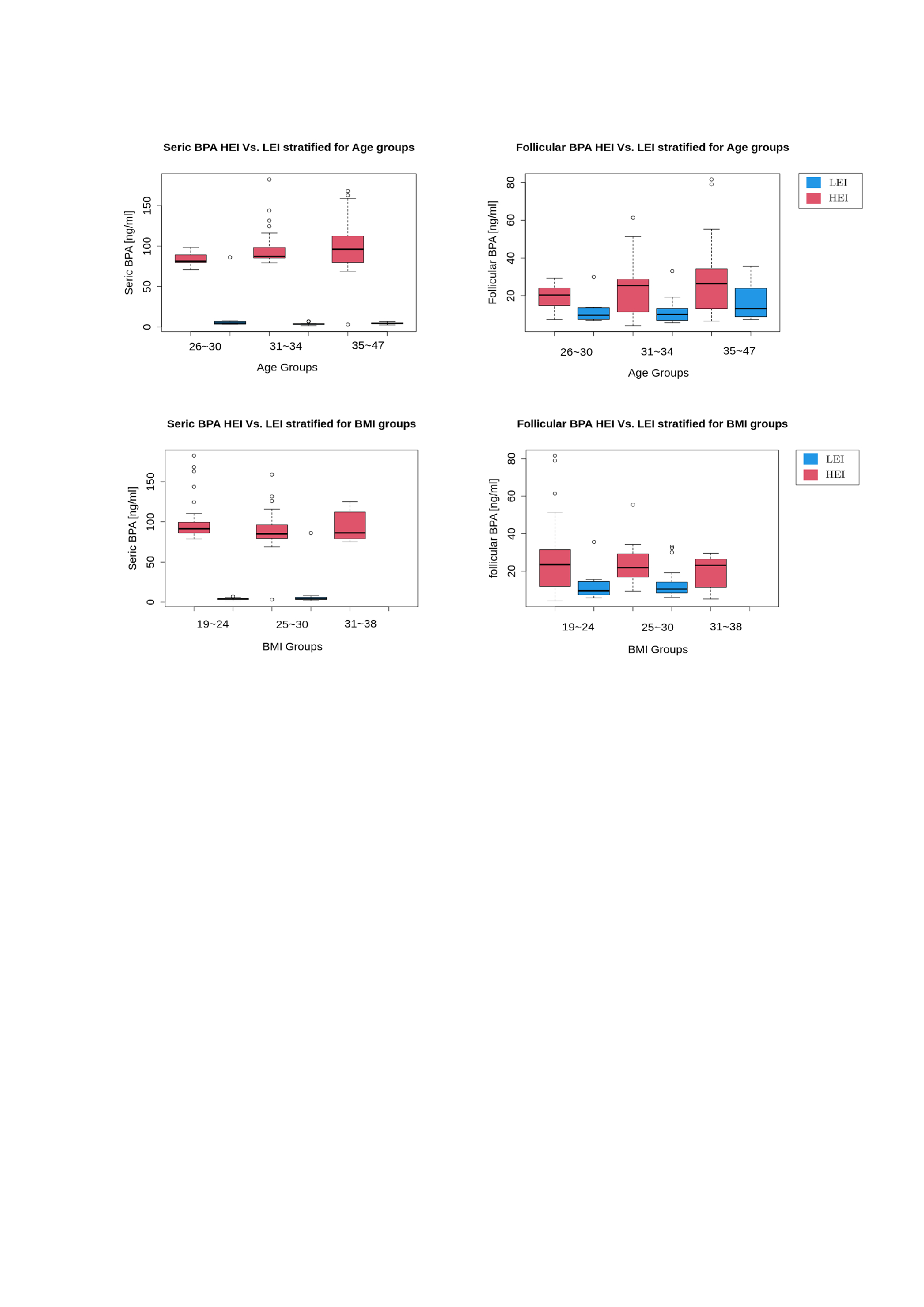

Supplement: Supplementary Figure 1 — Boxplots of numerical variables that resulted with significant differences in means, stratified per Age and BMI per zone (two tailed Wilcoxon test shown in Table 2 ). [file Image1.jpeg]
